# *In silico* screening of ethyl 4-[(E)-(2-hydroxy-4-methoxyphenyl)methyleneamino]benzoate and some of its derivatives for their NLO activities using DFT

**DOI:** 10.1098/rsos.220430

**Published:** 2023-01-11

**Authors:** Dinyuy Emmanuel Kiven, Nyiang Kennet Nkungli, Stanley Numbonui Tasheh, Julius Numbonui Ghogomu

**Affiliations:** ^1^ Department of Chemistry, Faculty of Science, The University of Bamenda, P.O. Box 39, Bambili-Bamenda, Cameroon; ^2^ Department of Chemistry, Faculty of Science, University of Dschang, Research Unit of Noxious Chemistry and Environmental Engineering, P.O. Box 67, Dschang, Cameroon

**Keywords:** ethyl 4-[(e)-(2-hydroxy-4-methoxyphenyl)methyleneamino]benzoate, hyperpolarizability, substitution effects, nonlinear optical, density functional theory

## Abstract

The nonlinear optical (NLO) properties of ethyl 4-[(*E*)-(2-hydroxy-4-methoxyphenyl)methyleneamino]benzoate (EMAB) and some of its derivatives are investigated herein using the density functional theory (DFT) and time-dependent (TD)-DFT methods. The density functionals B3LYP, CAM-B3LYP, M06-2X and *ω*B97XD, and basis sets 6-31 + G**, 6-311 + + G** and Def2-TZVPP have been used. From the results, EMAB and its substituted derivatives studied are promising candidates for NLO materials. In all cases, the static first and second hyperpolarizabilities (31.7–86.5 × 10^−30^ and 84.4–273 × 10^−36^ electrostatic units (esu), respectively) and the frequency-dependent NLO properties are found to be significantly larger (about 43–103 and 28–76 times greater) than those of the NLO prototypical molecule, *para*-nitroaniline. Furthermore, the maximum absorption wavelengths of the molecules fall within the UV region of the electromagnetic spectrum. Relative to EMAB, the derivatives have shown improved transparency–nonlinearity trade-offs. Natural bond orbital (NBO) and density of states (DOS) analyses herein revealed effective charge transfer within the molecules studied, especially those with stronger electron donors than that in EMAB (methoxy group). Among the molecules studied, the derivative obtained by substituting EMAB's methoxy group with the pyrrolyl group was found to exhibit the best NLO properties. Conclusively, the NLO activities of EMAB can be significantly improved through the substitution of its methoxy group with stronger electron donors.

## 1. Introduction

Nonlinear optical (NLO) materials are attracting much attention in recent times due to their fascinating applications in diverse fields of advanced technology, especially in optoelectronic and photonic technologies, where light is used as an information carrier [1–4]. Applications of NLO materials include their usage in modern communication technologies, optical switches and sensors, harmonic generators, modulators, optical computing, optical data processing, all-optical switching, optical data storage, optical limiting effects and holography [3,5–10]. Researchers are tirelessly devising new strategies for designing high-performance optoelectronic materials in view of achieving higher NLO responses with good optical transparencies [11]. It is believed that in the near future, light as an information carrier will be used in many more technologies [12]. Therefore, the search for new NLO materials and the repurposing of the older ones is imperative. In the past few decades, nearly all NLO materials comprised inorganic crystals. In recent times, there has been a paradigm shift toward organic electronic materials due to their higher NLO responses, as opposed to their inorganic counterparts [1,6,13,14]. Moreover, their synthesis is cost-effective and they also easily grow into high-quality crystals. Furthermore, not only are their molecular structures easily modifiable, they are easily incorporated into thin films [9,15,16].

Efficient NLO materials usually comprise molecules containing electron donor (D) and electron acceptor (A) groups connected by a π-conjugated bridge. Such molecules are described as push-pull or D-π-A molecules [8,17–19]. Intramolecular charge transfer between D and A in these push-pull molecules is responsible for their NLO activities [5,7,17,20,21]. This shift in electron density, which may result in second-order and/or third-order polarizability, is an important property of NLO materials [22,23]. The NLO responses of materials are often improved through structural and functional group modifications, typically by incorporating stronger electron donor/acceptor groups and/or modifying the nature/length of the π spacer [1,8,10,11,24].

**Figure 1.**
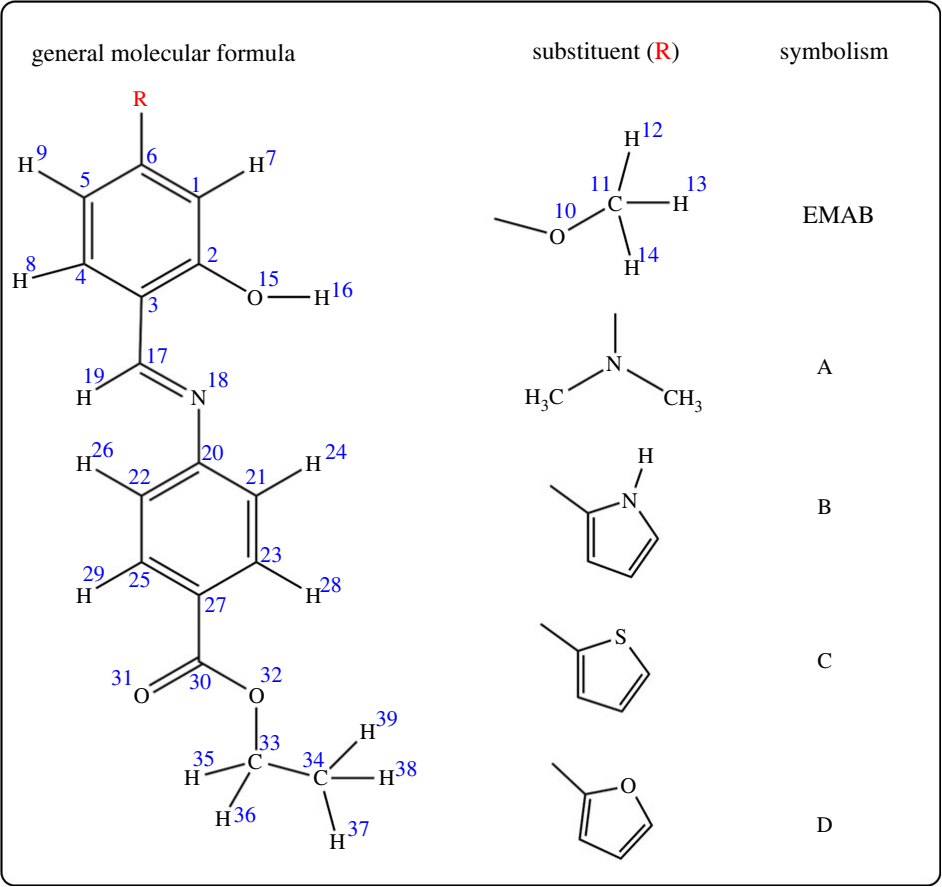
Molecular structure of ethyl 4-[(*E*)-(2-hydroxy-4-methoxyphenyl)methyleneamino]benzoate (EMAB) and some of its derivatives with electron-donating groups: A = dimethyl amino group, B = pyrrolyl group, C = thiophenyl group and D = furanyl group.

Schiff bases, which are compounds with the general formula R_1_R_2_C=N−R_3_, where R represents an alkyl or aryl group, exhibit significant intramolecular charge transfer [7], a possible reason for which they are often used as thin-film organic solar fluorescence materials as well as in organic light-emitting diodes and optical sensors [19]. As such, they are potential candidates for NLO applications, especially when composed of strong electron donor and/or acceptor groups [23,25]. According to literature survey, the Schiff base molecule shown in figure 1, ethyl 4-[(*E*)-(2-hydroxy-4-methoxyphenyl)methyleneamino]benzoate (EMAB) is planar, exhibits antimicrobial properties and some degree of hydrogen bonding [26]. As can be seen from figure 1, EMAB seemingly has a D-π-A structure with the structural moieties -OCH_3_ and -COOC_2_H_5_ acting as electron donor and acceptor, respectively. There is therefore a significant possibility of electron shift from one part of the molecule (donor) to the other (acceptor), making the molecule dipolar. The structure of EMAB therefore announces its potential as a good nonlinear optical chromophore. EMAB is thus expected to exhibit significant NLO properties that can be enhanced via substitution with stronger acceptor and/or donor groups, but this has not been investigated to date, to the best of our knowledge. Experimental or *in silico*-assisted studies in this perspective are likely to provide novel and fast-response NLO materials. Indeed, computational chemistry methods, particularly the density functional theory (DFT), have been very successful in this regard [4,6,8]. This method has therefore been adopted because it is faster than experimental and other theoretical methods, but yet yields similar results.

In the present work, the NLO susceptibilities and properties of EMAB alongside some of its derivatives with stronger electron donor groups have been investigated using the DFT method. The choice of the donor groups herein (displayed in figure 1) was guided by previous works in which they were found to improve intramolecular charge transfer (ICT), thereby increasing the NLO activities of the molecules studied therein [4,20,25]. Moreover, donor groups containing electronegative atom(s) linked to π-conjugated systems were preferred herein because they often lead to higher NLO activities [3,27–29]. Different levels of theory were used to calculate the static and dynamic first and second hyperpolarizabilities in the form of second harmonic generation (SHG), electro-optic Pockels effect (EOPE), the optical Kerr effect (OKE) and direct current SHG (DCSHG), which is also known as electric-field induced SHG (EFISHG), for comparison. In addition, the vector component of the dynamic first hyperpolarizability, *β*_vec_, which is usually the measured quantity in EFISHG experiments, was also calculated [18].

## 2. Computational details

All quantum chemical calculations were carried out using the Gaussian 09 Rev. D.01 program package [30]. Input files were prepared with the GaussView 6.0.16 program [31]. All post-processing of results was carried out with Multiwfn [32], Chemcraft [33] and GaussView 6.0.16 [31]. Conformational analysis to determine the most stable conformer of EMAB was performed using relaxed potential energy surface (PES) scans about the dihedral angles *ϕ*_1_ (C2-C3-C17-H19) and *ϕ*_2_ (C17-N18-C20-C22) at the B3LYP-D3(BJ)/6-31 + G** level of theory from 0 to 360° in 18 steps of 20° each. The structure corresponding to the global minimum on the PES scan was selected as the most stable conformer of EMAB. This lowest-energy conformer was further optimized and frequency calculations performed at B3LYP/6-311 + + G** and M06-2X/6-311 + + G** levels of theory. Furthermore, its derivatives (shown in figure 1) were optimized at the former level of theory. Analysis of the harmonic vibrational frequencies in all cases revealed no imaginary frequencies, ascertaining that the structures were true minima on their potential energy surface.

The global hybrid functional M06-2X (with 54% Hartree–Fock (HF) exchange [34]) was used along with two range-separated functionals *ω*B97-XD (with 22% HF exchange [35]) and CAM-B3LYP (with 19% HF exchange at short range and 65% HF exchange at long range [36]) to compute the NLO susceptibilities and properties herein. These functionals were employed together with the Pople-style and Ahlrichs basis sets, i.e. 6-311 + + G** [37,38] and Def2-TZVPP [39], respectively. M06-2X was chosen owing to its effectiveness in modelling non-covalent interactions and NLO properties of organic molecules [34,40]. Indeed, M06-2X is proficient and reliable in varied computational studies [10]. Although the global hybrid functionals have been popularly used to predict NLO properties, they suffer from limitations such as self-interaction error and incomplete description of long-range dispersion interactions [41]. The range-separated functionals were therefore used herein to complement the results obtained with the global hybrid functional, M06-2X. Moreover, global hybrid functionals have been reported to sometimes overestimate hyperpolarizabilities [17,42,43]. It must be pointed out that in all calculations, the restricted Kohn–Sham formalism (RKS) was adopted because all molecules studied are closed-shell systems, and all computations were performed in the gas phase. Furthermore, the long-range dispersion interactions were incorporated for functionals that lack an inbuilt dispersion correction (B3LYP and CAM-B3LYP) using the Grimme's dispersion correction, D3 [44]. To predict the UV-Vis electronic absorption wavelengths of the NLO chromophores, the time-dependent variant of the DFT method (TD-DFT) was used.

When a molecular material is subjected to an external electric field, the induced polarizability (*P*) can be expressed as a power series in the electric field *F* as follows:

2.1 $$P_{x}=\underset{y}{\sum }\alpha_{xy}F_{y}+\underset{y\leq z}{\sum }\beta_{xyz}F_{y}F_{z}+\underset{y\leq z\leq l}{\sum }\gamma_{xyzl}F_{y}F_{z}F_{l}+\ldots \;,$$

where *α* is the linear polarizability tensor, *β* is the first order hyperpolarizability (i.e. second-order or quadratic polarizability) tensor, and *γ* is the second order hyperpolarizability (i.e. third-order or cubic polarizability) tensor. The subscripts *x*, *y* and *z* are tensor components in the *x, y* and *z* directions, since the external electric field is applied to the molecule with components along the *x*, *y* and *z* directions [10,45].

In this study, the electronic dipole moment, *μ*, average linear polarizability, *α*, and its anisotropy, Δ*α*, for the compounds studied were calculated according to the following equations, using the finite-field method as implemented in Gaussian 09 program [6]:

2.2 $$\mu ={\left(\mu_{x}^{2}+\mu_{y}^{2}+\mu_{z}^{2}\right)}^{1/2},$$

2.3 $$\alpha =\frac{1}{3}(\alpha_{xx}+\alpha_{yy}+\alpha_{zz})$$

2.4 $$\mathrm{and}\;\Delta \alpha =\frac{1}{\sqrt{2}}{\left[{\left(\alpha_{xx}-\alpha_{yy}\right)}^{2}+{\left(\alpha_{yy}-\alpha_{zz}\right)}^{2}+{\left(\alpha_{zz}-\alpha_{xx}\right)}^{2}+6\alpha_{xy}^{2}+6\alpha_{xz}^{2}+6\alpha_{yz}^{2}\right]}^{1/2}.$$

The magnitude of the total static first hyperpolarizability, *β*_tot_, calculated as shown in equation (2.5) is a measure of the ease of electron redistribution in a material, in response to an external electric field.

2.5 $$\beta_{\mathrm{tot}}={\left(\beta_{x}^{2}+\beta_{y}^{2}+\beta_{z}^{2}\right)}^{1/2},$$

where *β_x_* = (*β_xxx_* + *β_xyy_* + *β_xzz_*), *β_y_* = (*β_yyy_* + *β_yzz_* + *β_yxx_*), *β_z_* = (*β_zzz_* + *β_zxx_* + *β_zyy_*) and *β_x_*, *β_y_* and *β_z_* are the components of *β*_tot_ along the *x*, *y* and *z* axes, respectively [5,8,11,29,46].

The first hyperpolarizability is a third-rank tensor described by a 3 × 3 matrix. Kleinman's symmetry is often used to reduce the components of this matrix from 27 to 10, denoted: *β_xxx_*, *β_yxx_*, *β_xyy_*, *β_yyy_*, *β_zxx_*, *β_xyz_*, *β_zyy_*, *β_xzz_*, *β_yzz_* and *β_zzz_*. [1,47]. It is important to note that the component *β_xyz_* is negligible and thus often left out, resulting to

$$\beta_{\mathrm{tot}}={\left[{\left(\beta_{xxx}+\beta_{xyy}+\beta_{xzz}\right)}^{2}+{\left(\beta_{yyy}+\beta_{yzz}+\beta_{yxx}\right)}^{2}+{\left(\beta_{zzz}+\beta_{zxx}+\beta_{zyy}\right)}^{2}\right]}^{1/2}.$$

The vector component of the first hyperpolarizability, as well as the second hyperpolarizability, were calculated as shown in equations (2.6) and (2.7) [3,28,45,48].

2.6 $$\beta_{\mathrm{vec}}={\left(\beta_{x}^{2}+\beta_{y}^{2}+\beta_{z}^{2}\right)}^{1/2}$$

and

2.7 $$\gamma =\frac{1}{15}\underset{ij=x,y,z}{\sum }\gamma_{iiij}+\gamma_{ijij}+\gamma_{ijji}.$$

Application of the Kleinman's symmetry reduces the components of *γ* in equation (2.7) to six components, used to calculate the overall second-order hyperpolarizability as shown in the following equation [9,10,15]:

2.8 $$\left\langle \gamma \right\rangle =\;1/5(\gamma_{xxxx}+\;\gamma_{yyyy}+\;\gamma_{zzzz}+\;2\gamma_{xxyy}\;+\;2\gamma_{xxzz}\;+\;2\gamma_{yyzz}).$$

Besides the static polarizability, *α*(0;0) static first hyperpolarizability, *β*(0;0, 0) and static second hyperpolarizability, *γ*(0;0, 0, 0) their frequency-dependent (dynamic) counterparts were calculated at the Nd:YAG laser wavelength (*λ*) of 1064 nm which corresponds to the frequency *ω* = 0.04282 arb. units, and at the green laser pointer wavelength 532 nm, corresponding to *ω* = 0.08564 arb. units.

## 3. Results and discussion

### 3.1. Conformational analysis

**Figure 2.**
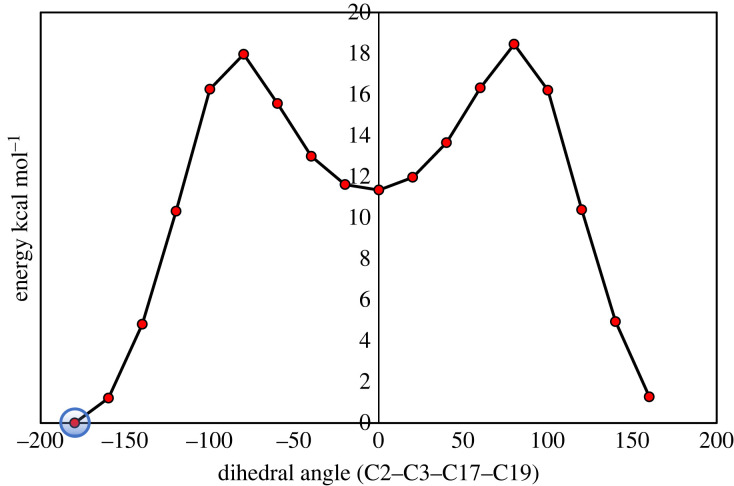
Potential energy profile of EMAB about *ϕ*_1_ (C2-C3-C17-H19) calculated at B3LYP-D3(BJ)/6-31 + G** level of theory (minimum energy = 6.2751 × 10^−6^ kcal mol^−1^).

**Figure 3.**
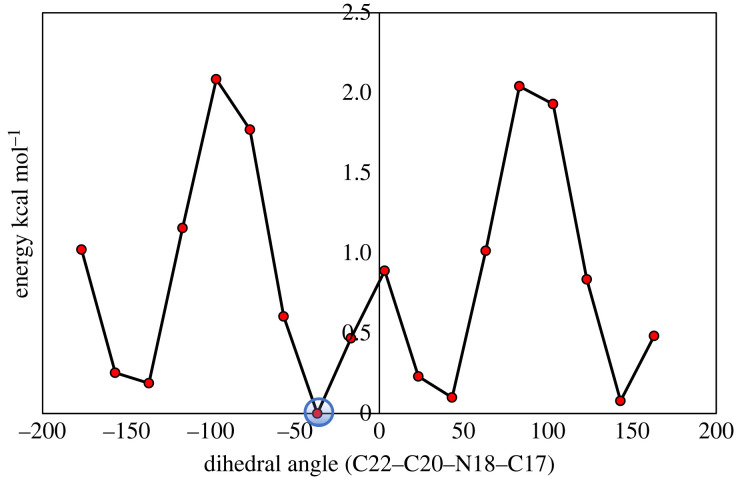
Potential energy profile of EMAB about *ϕ*_2_ (C17-N18-C20-C22) calculated at B3LYP-D3(BJ)/6-31 + G** level of theory (minimum energy = 6.2751 × 10^−6^ kcal mol^-1^).

As can be seen from figure 1, EMAB can undergo free rotation about the single bonds C3-C17 and C20-N18, implying that the molecule may adopt two or more stable conformational structures. It is desirable that *in silico* predictions be made based on the most stable conformer of the molecule. To determine the most stable conformer of EMAB, relaxed PES scans about the torsional angles *ϕ*_1_ (C2-C3-C17-H19) and *ϕ*_2_ (C17-N18-C20-C22) were performed as described earlier [49]. The PES scan curves (shown in figures 2 and 3) revealed the lowest-energy structure for rotation about *ϕ*_1_ at 6.2751 × 10^−6^ kcal mol^−1^ and that for rotation about *ϕ*_2_ at 6.2751 × 10^−6^ kcal mol^−1^.

The minimum energy point encircled on each PES curve corresponds to the most stable conformer. Interestingly, the two PES scans revealed the same conformer of EMAB as the most stable, judging from their nearly identical energies and three-dimensional structures. This conformer was therefore used in all subsequent calculations and analyses.

Selected geometric parameters of the most stable conformer of EMAB are presented in table 1, as obtained from geometry optimization at B3LYP/6-311 + + G** and M06-2X/6-311 + + G** levels of theory, alongside the X-ray diffraction values obtained from the literature [26].

**Table 1.** Some theoretical geometric parameters of EMAB computed at B3LYP/6-311 + + G** and M06-2X/6-311 + + G** levels of theory compared with experimental values.

geometric parameter	theoretical values		experimental values^a^
	B3LYP	M06-2X	
bond lengths (Å)			
O32-C33	1.450	1.449	1.445
O32-C30	1.354	1.353	1.343
O31-C30	1.219	1.219	1.21
O15-C2	1.343	1.342	1.349
O10-C6	1.359	1.359	1.363
O10-C11	1.425	1.424	1.425
N18-C20	1.404	1.402	1.411
N18-C17	1.297	1.296	1.284
C2-C1	1.394	1.393	1.386
C1-C6	1.398	1.397	1.382
bond angles (°)			
C30-O32-C33	117	116.7	116.8
O32-C33-C34	111	111.3	107.4
O32-C30-C27	112.5	112.4	112.4
O31-C30-C32	123.3	123.3	122.7
O31-C30-C27	124.1	124.2	124.9
C21-C20-N18	118	118	116.9
C22-C20-N18	122.9	122.9	125
C17-N18-C20	121.1	121.1	122
N18-C17-C3	122.4	122.3	122.3
O15-C2-C3	121.3	121.3	121.1
O15-C2-C1	118.6	118.8	118.3
O10-C6-C1	123.8	123.7	124.7
O10-C6-C5	115.4	115.4	114.5
C6-O10-C11	119.1	118.8	117.6
dihedral angles (°)			
C17-N18-C20-C22	−36.8	−36.0	—
C2-C3-C17-H19	−179.8	−179.9	—
C21-C20-N18-C17	145.6	145.5	—
N18-C17-C3-C4	179.2	179.3	—
O32-C30-C27-C25	−179.6	−179.1	—
C1-C6-O10-C11	−179.9	−179.9	—
hydrogen bond parameters			
O15-H16 (Å)	1.0	1.0	0.9
N18-H16 (Å)	1.7	1.7	1.9
O15-H16-N18	147.6	147.9	140.7

^a^The experimental values were obtained from [26].

**Figure 4.**
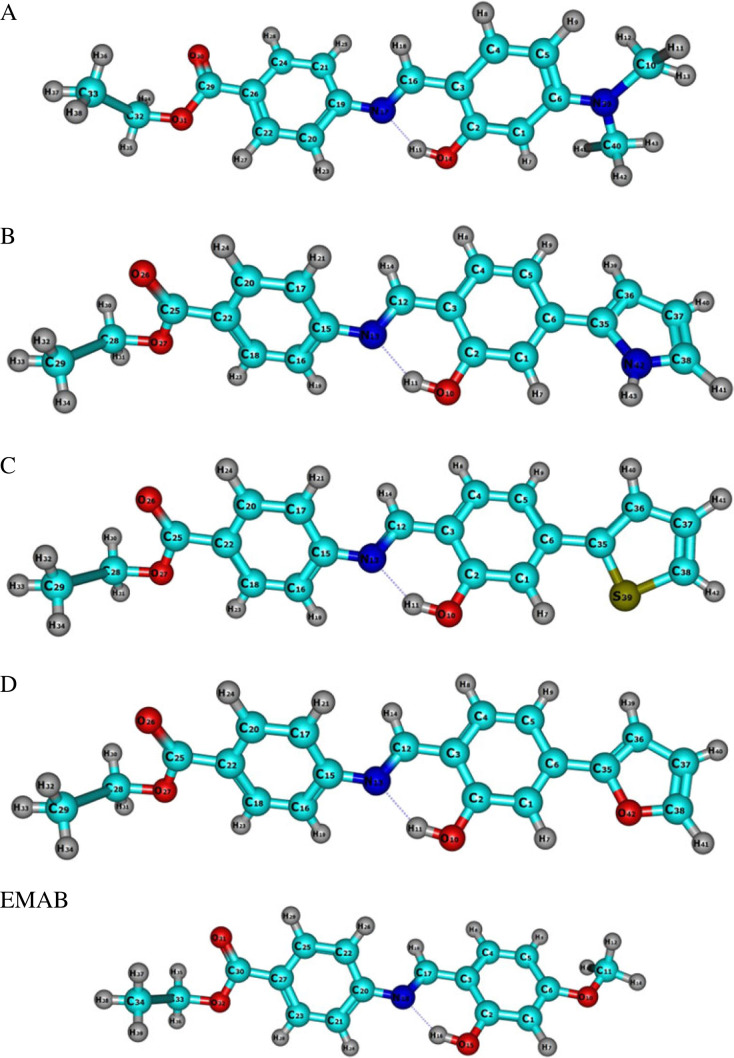
Optimized structures of EMAB and its derivatives at B3LYP-(D3)/6-311 + + G** level of theory.

It is clear from table 1 that nearly identical geometric parameters of EMAB are generated at both levels of theory. It can also be seen that the calculated sets of values are each in good agreement with the corresponding experimental values. To better compare the theoretical and experimental values, linear regression and correlation analyses were carried out, and the results presented in electronic supplementary material, figure S1. From this figure, we observe that larger *R*^2^ values were obtained for both geometrical parameters with the B3LYP functional (0.9868 and 0.9655) for bond lengths and bond angles respectively, compared with M06-2X functional, with *R*^2^ values 0.9867 and 0.9614 for bond lengths and bond angles respectively. Therefore, the structure of EMAB (and arguably its derivatives) optimized at the B3LYP-D3/6-311 + + G** level of theory had been more appropriate for further investigations. Accordingly, the linear and nonlinear optical property computations have been performed using B3LYP-optimized structures, depicted in figure 4. Molecular coordinates for optimized molecules studied are found in electronic supplementary material, tables S3–S7

Had the structure of EMAB been perfectly planar, the dihedral angle C17-N18-C20-C22 in table 1 would have been 0°, while the rest of the dihedral angles in the table would have been 180°. The observed moderate to slight deviations from these ideal values indicate that EMAB possesses some reasonable degree of planarity. Since planarity is a consequence of π-conjugation in a molecule, it follows that ICT between the electron donor and acceptor in EMAB is possible, and likely to yield a significant NLO response. The intramolecular hydrogen bond in EMAB and its derivatives (shown in figure 4) not only causes stabilization but also contributes to the planarity of the molecules. According to Jeffrey's hydrogen bond classification [50] the hydrogen bond interaction in EMAB is mainly electrostatic (with a bond angle of about 140° and bond length of about 1.9 Å) and is of moderate strength.

### 3.2. Nonlinear optical susceptibilities and properties

#### 3.2.1. Static polarizability isotropies (*α*), anisotropies (Δ*α*) and norm dipole moments (*μ*)

Linear polarizability plays a critical role in electronic charge distribution in a molecule. In response to an applied electric field, there is charge separation in a molecule that creates positive and negative poles. The extent of the charge separation depends on how loose or tight electrons are held [51]. The linear isotropic and anisotropic polarizabilities of all the molecules studied in this work are presented in table 2, as calculated at different levels of theory.

**Table 2.** Linear static polarizability isotropies (*α*) × 10^−24^, anisotropies (Δ*α*) × 10^−24^ and norm dipole moments (*μ*) × 10^−18^ electrostatic units (esu) of EMAB and its derivatives calculated at different levels of theory.

molecule	level of theory	*μ_x_*	*μ_y_*	*μ_z_*	*μ*	*α_xx_*	*α_yy_*	*α_zz_*	*α*	Δ*α*
EMAB	M06-2X/6-311 + + G**	1.86	−1.94	1.44	3.05	64.8	29.0	19.9	37.9	41.2
	M06-2X/Def2-TZVPP	1.99	−1.649	1.38	2.93	66.1	29.1	19.9	38.4	42.4
	CAM-B3LYP/6-311 + + G**	1.96	−1.96	1.47	3.14	65.3	29.4	20.1	38.2	38.2
	CAM-B3LYP/Def2-TZVPP	2.09	−1.66	1.41	3.02	66.4	29.2	19.9	38.5	42.7
	*ω*B97-XD/6-311 + + G**	1.97	−1.88	1.45	3.08	64.8	29.4	20.1	38.1	40.9
	*ω*B97-XD/Def2-TZVPP	2.10	−1.58	1.38	2.97	65.9	29.3	20.0	38.4	42.2
A	M06-2X/6-311 + + G**	−4.55	−7.29	1.53	4.86	74.8	31.5	21.9	42.7	49.0
	M06-2X/Def2-TZVPP	−4.59	−5.52	1.49	4.86	76.2	31.6	21.9	43.3	50.2
	CAM-B3LYP/6-311 + + G**	−4.59	−7.36	1.55	4.90	74.8	31.8	22.1	42.9	48.7
	CAM-B3LYP/Def2-TZVPP	−4.64	−5.51	1.49	4.90	75.9	31.7	21.9	43.2	50.0
	*ω*B97-XD/6-311 + + G**	−4.55	−6.83	1.54	4.86	74.1	31.8	22.1	42.7	47.9
	*ω*B97-XD/Def2-TZVPP	−4.6	−4.99	1.49	4.86	75.3	31.8	22.0	43.0	49.2
B	M06-2X/6-311 + + G**	0.93	−0.48	−0.29	1.09	84.8	33.4	22.9	47.1	57.4
	M06-2X/Def2-TZVPP	1.03	−0.66	−0.29	1.26	86.1	33.3	22.9	47.5	58.7
	CAM-B3LYP/6-311 + + G**	0.99	−0.47	−0.32	1.14	85.0	33.7	23.2	47.3	57.3
	CAM-B3LYP/Def2-TZVPP	1.09	−0.65	−0.31	1.31	86.1	33.4	22.8	47.5	58.7
	*ω*B97-XD/6-311 + + G**	0.92	−0.54	−0.30	1.11	83.9	33.7	23.2	47.0	56.3
	*ω*B97-XD/Def2-TZVPP	1.01	−0.72	−0.29	1.27	85.1	33.5	22.9	47.2	57.6
C	M06-2X/6-311 + + G**	0.96	1.01	−1.02	1.73	83.5	36.1	22.9	47.5	55.3
	M06-2X/Def2-TZVPP	1.06	0.83	−0.99	1.67	84.9	36.2	22.7	47.9	56.6
	CAM-B3LYP/6-311 + + G**	1.05	1.03	−1.01	1.81	83.7	36.5	23.1	47.7	55.2
	CAM-B3LYP/Def2-TZVPP	1.15	0.84	−1.01	1.74	84.9	36.2	26.6	47.9	56.8
	*ω*B97-XD/6-311 + + G**	1.71	1.50	−1.20	2.56	74.4	35.2	22.7	44.1	46.8
	*ω*B97-XD/Def2-TZVPP	1.60	−0.82	−0.98	2.03	84.1	36.3	22.7	47.7	55.9
D	M06-2X/6-311 + + G**	1.50	1.10	−1.20	2.22	82.0	33.2	21.8	45.7	55.5
	M06-2X/Def2-TZVPP	1.60	0.89	−1.10	2.14	83.5	33.3	21.8	46.2	56.9
	CAM-B3LYP/6-311 + + G**	1.60	1.10	−1.20	2.31	82.3	33.6	21.9	46.0	55.4
	CAM-B3LYP/Def2-TZVPP	1.70	0.90	−1.20	2.22	83.6	33.4	21.7	46.3	57.0
	*ω*B97-XD/6-311 + + G**	1.50	1.10	−1.20	2.22	81.4	33.6	22.0	45.7	54.6
	*ω*B97-XD/Def2-TZVPP	1.60	0.80	−1.10	2.12	82.8	33.5	21.8	46.0	56.1
PNA	M06-2X/6-311 + + G**	−0.90	6.10	0.00	6.18	9.8	18.3	12.7	13.6	7.5
	M06-2X/Def2-TZVPP	−0.80	5.90	0.00	6.02	9.6	18.4	12.9	13.6	7.7
	CAM-B3LYP/6-311 + + G**	−0.90	6.00	0.00	6.10	9.9	18.5	12.9	13.8	7.5
	CAM-B3LYP/Def2-TZVPP	−0.80	5.90	0.00	5.95	9.5	18.3	12.8	13.6	7.8
	*ω*B97-XD/6-311 + + G**	−0.90	5.90	0.00	6.06	9.9	18.5	12.9	13.8	7.5
	*ω*B97-XD/Def2-TZVPP	−0.80	5.80	0.00	5.90	9.5	18.4	12.9	13.6	7.8

**Figure 5.**
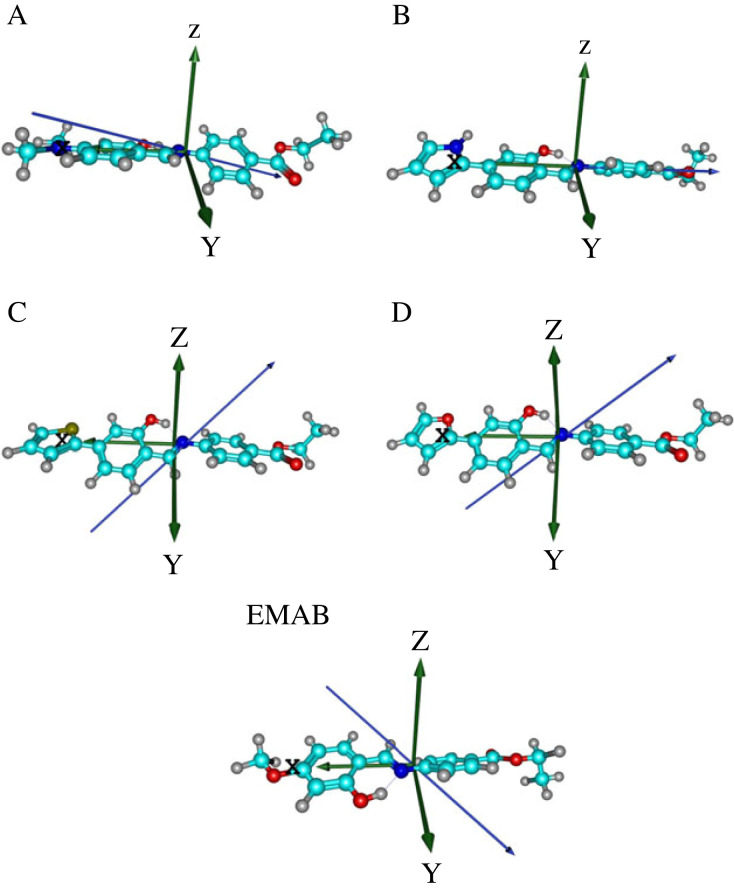
Optimized structures of EMAB and its derivatives at the B3LYP/6-311G** level of theory. The dipole moment vector is shown in blue and the coordinate axes are in green.

Among the polarizability tensor components, the values of *α_xx_* are the largest, indicating that polarizability occurs mainly along the *x*-axis of the molecules, whose Cartesian coordinates originate from the centre of mass. Molecule C exhibits the largest isotropic polarizability of about 47.5 × 10^−24^ electrostatic units (esu), as calculated using the M06-2X and CAM-B3LYP functionals in conjunction with the basis set Def2-TZVPP. At these levels of theory, the largest anisotropic polarizability of 58.7 × 10^−24^ esu is obtained for B. It is also clear from this table that the linear polarizabilities calculated at the foregoing levels of theory show a similar trend: C > B > D > A > EMAB. The noticeable discrepancies between the isotropic and anisotropic polarizability values in all cases at all levels of theory indicate that polarizability is dependent on the orientation of the applied electric field. Also presented in table 2 are the norm dipole moments of the investigated molecules as calculated at all levels of theory. The dipole moment vectors of the molecules studied are shown in figure 5.

It can be seen from the vector orientations (figure 5) and *x*-components of *μ* (table 2) that the vector component of the dipole moment of B lies almost entirely along its *x*-axis, which is aligned with the *π*-conjugated bridge. Consequently, ICT is expected to be more significant in B than in all the other molecules studied. Generally, the dipole moment vectors of EMAB's derivatives are found to be more aligned with the *π*-conjugated bridge than is the case with the parent molecule.

#### 3.2.2. Static first and second hyperpolarizabilities

Hyperpolarizability is a physical quantity that measures the susceptibility of a molecule to undergo NLO activities in response to an externally applied static or dynamic electric field. In particular, the first hyperpolarizability (*β*_tot_) has attracted much attention from both experimental and theoretical perspectives because of its vital role in the design of novel NLO materials for various applications. The static hyperpolarizability measures a molecule's susceptibility to NLO activities based on a static electric field with frequency *ω* = 0. Consequently, the static first and second hyperpolarizabilities are denoted *β*(0;0,0) and *γ*(0;0,0,0), respectively. The calculated values of *β*(0;0,0) and *γ*(0;0,0,0) in this work for all molecules studied are presented in tables 3 and 4, alongside their tensor components along the *x*, *y* and *z* axes of the molecules.

**Table 3.** Static total quadratic hyperpolarizability and its *x*, *y* and *z* components for EMAB and its derivatives.

molecule	level of theory	*β_x_*	*β_y_*	*β_z_*	*β*_tot_ × 10^−30^ esu
EMAB	M06-2X/6-311 + + G**	35.1	5.2	−1.5	35.5
	M06-2X/Def2-TZVPP	34.7	4.5	−1.3	35.0
	CAM-B3LYP/6-311 + + G**	34.4	5.5	−1.7	34.8
	CAM-B3LYP/Def2-TZVPP	33.4	4.7	−1.5	33.8
	*ω*B97-XD/6-311 + + G**	32.2	5.3	−1.7	32.7
	*ω*B97-XD/Def2-TZVPP	31.3	4.6	−1.5	31.7
A	M06-2X/6-311 + + G**	−67.7	4.6	0.6	67.8
	M06-2X/Def2-TZVPP	−66.6	3.9	0.8	66.7
	CAM-B3LYP/6-311 + + G**	−63.3	4.8	0.3	63.4
	CAM-B3LYP/Def2-TZVPP	−61.4	3.9	0.4	61.6
	*ω*B97-XD/6-311 + + G**	−58.8	4.7	0.2	59.0
	*ω*B97-XD/Def2-TZVPP	−57.0	3.9	0.4	57.2
B	M06-2X/6-311 + + G**	85.1	−6.4	1.3	85.3
	M06-2X/Def2-TZVPP	86.3	−6.0	1.4	86.5
	CAM-B3LYP/6-311 + + G**	79.8	−6.3	1.33	80.0
	CAM-B3LYP/Def2-TZVPP	80.5	−5.9	1.5	80.7
	*ω*B97-XD/6-311 + + G**	72.1	−6.1	1.3	72.3
	*ω*B97-XD/Def2-TZVPP	72.7	−5.6	1.4	73.0
C	M06-2X/6-311 + + G**	50.6	−3.6	1.9	50.8
	M06-2X/Def2-TZVPP	51.7	−3.7	1.6	51.9
	CAM-B3LYP/6-311 + + G**	47.6	−3.7	1.9	47.8
	CAM-B3LYP/Def2-TZVPP	51.7	−3.7	1.6	51.9
	*ω*B97-XD/6-311 + + G**	27.0	−2.8	1.5	27.1
	*ω*B97-XD/Def2-TZVPP	43.5	−3.6	1.6	43.7
D	M06-2X/6-311 + + G**	63.9	−5.1	1.4	64.1
	M06-2X/Def2-TZVPP	65.3	−4.8	1.4	65.5
	CAM-B3LYP/6-311 + + G**	59.2	−5.0	1.5	59.4
	CAM-B3LYP/Def2-TZVPP	60.0	−4.6	1.5	60.2
	*ω*B97-XD/6-311 + + G**	54.4	−4.9	1.5	54.7
	*ω*B97-XD/Def2-TZVPP	55.2	−4.5	1.4	55.4
PNA	M06-2X/6-311 + + G**	0.2	0.8	0.0	0.8
	M06-2X/Def2-TZVPP	0.2	0.6	0.0	0.7
	CAM-B3LYP/6-311 + + G**	0.2	0.7	0.0	0.7
	CAM-B3LYP/Def2-TZVPP	0.3	0.5	0.0	0.5
	*ω*B97-XD/6-311 + + G**	0.2	0.6	0.0	0.6
	*ω*B97-XD/Def2-TZVPP	0.2	0.5	0.0	0.5

**Table 4.** Static average cubic hyperpolarizability and its *x*, *y* and *z* components for EMAB and its derivatives.

molecule	level of theory	*γ_x_*	*γ_y_*	*γ_z_*	〈*γ*〉 × 10^−36^ esu
EMAB	M06-2X/6-311 + + G**	94.3	4.25	4.49	103
	M06-2X/Def2-TZVPP	90.5	1.70	1.57	93.8
	CAM-B3LYP/6-311 + + G**	92.9	4.67	4.87	102
	CAM-B3LYP/Def2-TZVPP	88.4	1.63	1.50	91.5
	*ω*B97-XD/6-311 + + G**	84.8	4.61	4.87	94.3
	*ω*B97-XD/Def2-TZVPP	81.0	1.78	1.57	84.4
A	M06-2X/6-311 + + G**	154	5.80	6.52	166
	M06-2X/Def2-TZVPP	147	2.07	2.25	151
	CAM-B3LYP/6-311 + + G**	145	6.35	7.00	159
	CAM-B3LYP/Def2-TZVPP	138	2.03	2.16	142
	*ω*B97-XD/6-311 + + G**	130	6.05	6.75	143
	*ω*B97-XD/Def2-TZVPP	124	2.19	2.23	129
B	M06-2X/6-311 + + G**	262	5.1	6.0	273
	M06-2X/Def2-TZVPP	252	1.6	1.7	255
	CAM-B3LYP/6-311 + + G**	248	5.6	6.6	260
	CAM-B3LYP/Def2-TZVPP	237	1.4	1.6	240
	*ω*B97-XD/6-311 + + G**	218	5.5	6.6	230
	*ω*B97-XD/Def2-TZVPP	208	1.6	1.7	211
C	M06-2X/6-311 + + G**	216	5.2	5.8	227
	M06-2X/Def2-TZVPP	208	1.7	1.6	211
	CAM-B3LYP/6-311 + + G**	207	5.5	6.2	219
	CAM-B3LYP/Def2-TZVPP	198	1.6	1.5	201
	*ω*B97-XD/6-311 + + G**	106	3.8	5.5	116
	*ω*B97-XD/Def2-TZVPP	174	1.8	1.8	178
D	M06-2X/6-311 + + G**	228	4.7	5.6	238
	M06-2X/Def2-TZVPP	221	1.7	1.6	224
	CAM-B3LYP/6-311 + + G**	216	5.1	6.1	227
	CAM-B3LYP/Def2-TZVPP	209	1.5	1.5	212
	*ω*B97-XD/6-311 + + G**	192	5.1	6.1	203
	*ω*B97-XD/Def2-TZVPP	186	1.7	1.6	189
PNA	M06-2X/6-311 + + G**	1.7	3.4	1.8	6.9
	M06-2X/Def2-TZVPP	0.3	2.2	0.8	3.4
	CAM-B3LYP/6-311 + + G**	1.9	3.7	1.9	7.5
	CAM-B3LYP/Def2-TZVPP	0.3	2.3	0.8	3.4
	*ω*B97-XD/6-311 + + G**	1.9	3.6	1.9	7.5
	*ω*B97-XD/Def2-TZVPP	0.3	2.3	0.9	3.5

According to the results, the predicted *β*(0;0,0) and *γ*(0;0,0,0) for EMAB and its derivatives are remarkably larger than those of the prototype NLO molecule, *para*-nitroaniline (PNA), at all levels of theory considered. This is indicative of their high susceptibility to exhibit significant NLO activities. Strictly speaking, the values of *β*_tot_ for B, A, D, C and EMAB are larger than those of PNA by factors of approximately 103, 82, 77, 61 and 43, respectively. Similarly, their 〈*γ*〉 values are about 76, 67, 63, 50 and 28 times larger than those of PNA. Based on the foregoing observations, the derivatives of EMAB are promising dipolar (possessing electron donor and acceptor ends) NLO chromophores that are potentially applicable in optoelectronics and all-optical technologies.

From the values in tables 3 and 4, it can be seen that the values of *β*_tot_ generally increase in the order: EMAB < A < C < D < B; likewise those of 〈*γ*〉 increase in the order: EMAB < A < C < D < B. Therefore, the second- and third-order NLO activities of all EMAB's derivatives bearing stronger electron donors are expected to be significantly higher than those of the parent molecule. Clearly, EMAB's high NLO activities, as predicted in this work, can generally be further improved via substitution with stronger electron donors, such as those investigated herein. It is also clear from our results that *β_xxx_* and *β_x_* are the principal components of *β*(0;0,0), as well as *γ_xxx_* and *γ_x_* are the major components of *γ*(0;0,0,0) in all cases. This suggests that ICT in the molecules is dominant along the *x*-direction, along which lies the bulk of each molecule's *π*-conjugated backbone. The values of *β_xxx_* and *γ_xxxx_*, together with other tensor components are provided in electronic supplementary material, tables S1 and S2, respectively. Interestingly, similar trends in the results have been obtained at all levels of theory, albeit relatively higher values predicted by the M06-2X (global hybrid) functional, whereas those predicted by the *ω*B97-XD (range separated) functional are relatively lower.

#### 3.2.3. The vector component of the first hyperpolarizability ($\beta_{\mathrm{vec}}$)

The computed vector components of the dynamic first hyperpolarizability (*β*_vec_), i.e. the component of the *β_ijk_* tensor projected along the dipole moment axis, are presented in table 5 as calculated at two laser wavelengths: *λ*_1_ *=* 532 nm (*ω* = 0.08568) and *λ*_2_ = 1064 nm (*ω* = 0.04282 arb. units).

**Table 5.** Vector components of the quadratic polarizabilities of EMAB and its derivatives at 532 and 1064 nm.

molecule	level of theory	wavelength 532 nm				wavelength 1064 nm			
		$\beta_{x}$	$\beta_{y}$	$\beta_{z}$	$\beta_{\mathrm{vec}}$×$10^{-30}\;$esu	$\beta_{x}$	$\beta_{y}$	$\beta_{z}$	$\beta_{\mathrm{vec}}$×$10^{-30}\;$esu
EMAB	M06-2X/6311 + + G**	1670	−173	56.6	1160	58.2	7.3	−2.2	29.9
	M06-2X/Def2-TZVPP	1980	-215	67.8	1500	57.9	6.3	-1.9	34.8
	CAM-B3LYP/6-311 + + G**	609	-53.4	17.2	423	56.8	7.6	-2.4	29.7
	CAM-B3LYP/Def2-TZVPP	784	-73.9	22.7	595	55.5	6.6	-2.1	34
	*ω*B97-XD/6-311 + + G**	420	-42.2	13.8	301	52.2	7.5	-2.3	27.7
	*ω*B97-XD/Def2-TZVPP	514	-55.4	17.4	401	51	6.5	-2.1	31.7
A	M06-2X/6-311 + + G**	508	22.5	-23.4	-487	-132	6.5	2.0	123
	M06-2X/Def2-TZVPP	505	24.1	-22.2	-487	-130	5.5	2.2	123
	CAM-B3LYP/6-311 + + G**	508	23.7	-23.4	-487	-120	6.8	1.4	112
	CAM-B3LYP/Def2-TZVPP	494	25.3	-21.7	-477	-117	5.6	1.6	111
	*ω*B97-XD/6-311 + + G**	820	49.6	-38.8	−789	−108	6.6	1.1	101
	*ω*B97-XD/Def2-TZVPP	657	41.8	−30.7	−636	−106	5.6	1.3	99.8
B	M06-2X/6-311 + + G**	−461	17.2	−12.5	−398	183	−11.6	2.6	161
	M06-2X/Def2-TZVPP	−489	14.2	−10.1	−406	184	−11.0	2.7	156
	CAM-B3LYP/6-311 + + G**	−316	18.8	−12.7	−278	168	−11.0	2.6	150
	CAM-B3LYP/Def2-TZVPP	−336	16.4	−10.8	−286	168	−10.4	2.7	145
	*ω*B97-XD/6-311 + + G**	−330	11.2	−10.4	−276	145	−10.1	2.3	125
	*ω*B97-XD/Def2-TZVPP	−350	9.0	−8.8	−281	145	−9.5	2.4	120
C	M06-2X/6-311 + + G**	−345	12.2	−2.86	−183	97.9	−6.0	3.1	49.0
	M06-2X/Def2-TZVPP	−363	10.8	−2.26	−224	99.2	−5.8	2.8	58.4
	CAM-B3LYP/6-311 + + G**	−200	16.0	−3.90	−104	91.0	−5.8	3.2	47.4
	CAM-B3LYP/Def2-TZVPP	−204	14.6	−3.80	−125	91.4	−5.7	2.8	55.7
	*ω*B97-XD/6-311 + + G**	−57	1.08	−1.88	−3.68	41.6	−3.5	2.0	25.0
	*ω*B97-XD/Def2-TZVPP	−226	5.74	−1.94	−143	79.6	−5.4	2.6	47.6
D	M06-2X/6-311 + + G**	−427	13.9	−7.7	−282	127	−8.2	2.5	81.7
	M06-2X/Def2-TZVPP	−458	10.3	−6.3	−331	130	−7.9	2.4	91.4
	CAM-B3LYP/6-311 + + G**	−283	16.8	−8.6	−184	115	−7.9	2.5	74.9
	CAM-B3LYP/Def2-TZVPP	−305	1.40	−7.5	218	117	−7.4	2.2	83.2
	*ω*B97-XD/6-311 + + G**	−295	8.17	−6.4	−198	102	−7.6	2.4	66.7
	*ω*B97-XD/Def2-TZVPP	104	−7.10	2.3	73.9	104	−7.1	2.3	73.9
PNA	M06-2X/6-311 + + G**	0.9	6.3	0.0	6.1	0.3	1.2	0.0	1.1
	M06-2X/Def2-TZVPP	0.7	5.7	0.0	5.5	0.3	0.9	0.0	0.9
	CAM-B3LYP/6-311 + + G**	0.9	6.4	0.0	6.2	0.2	0.9	0.0	0.9
	CAM-B3LYP/Def2-TZVPP	0.7	5.9	0.0	5.7	0.3	0.8	0.0	0.7
	*ω*B97-XD/6-311 + + G**	0.8	6.0	0.0	5.8	0.2	0.9	0.0	0.8
	*ω*B97-XD/Def2-TZVPP	0.7	5.6	0.0	5.5	0.3	0.7	0.0	0.6

At both wavelengths, the *β*_vec_ values of the investigated molecules are significantly larger than those of PNA, which is indicative of their likelihood to act as interesting NLO materials. At the Nd:YAG laser wavelength, 1064 nm, the vector components of the dynamic first hyperpolarizability of all the molecules are positive, and are therefore parallel to the dipole moment vector, whereas at laser wavelength 532 nm, the *β*_vec_ values for the molecules A–D are negative, and are thus antiparallel to the dipole moment vector. It is evident from table 5 that the magnitude of *β*_vec_ decreases drastically as the green laser wavelength doubles (as 532 nm changes to 1064 nm), or as the frequency of the laser, *ω* = 0.08568, drops to half its value. An examination of this table reveals that substitution has a minimal effect on the *β*_vec_ value of EMAB at 532 nm, whereas a significant effect is observed at 1064 nm. At the latter wavelength, the *β*_vec_ values calculated at all levels of theory follow the ranking: EMAB < C < D < A < B, and are also found to be larger than those of PNA by factors of approximately 36, 63, 99, 133 and 170, respectively. These results suggest that the molecules under investigation, most especially the derivatives, are promising materials for the fabrication of optoelectronic and photonic devices.

It is clear from table 5 that substituting the methoxy group in EMAB with the pyrrolyl group significantly improves the NLO susceptibility of the molecule. Unexpectedly, substitution with the thiophenyl group that possesses a higher electron density and more delocalized electrons [52] results in lower hyperpolarizability than that obtained when the substituents (donors) are pyrrolyl and furanyl. The nitrogen atom in pyrrole can conjugate with π-electrons in the ring, thus increasing the electron density since pyrrole is a π excessive heterocycle. Moreover, pyrrole is more susceptible to electrophilic attack due to the greater electron-releasing ability of neutral trivalent nitrogen, and the accompanying greater stability of a positive charge on tetravalent nitrogen [52,53], more readily than the oxygen atom in furan, which is more electronegative [53]. Furthermore, the *σ* and π complexes for attack by electrophiles in pyrrole at 2 position are stabilized by a carbenium-iminium mesomerism which reduces ΔH for the rate-determining step. The opposite takes place in furan [52]. The higher reactivity of furan, compared with thiophene, may be due to the smaller mesomeric effect of the sulfur atom compared with that of oxygen.

As earlier noted, the values of static *β*_tot_, static 〈*γ*〉 and dynamic *β*_vec_ calculated at six levels of theory follow similar trends. Since the global hybrid functionals tend to overestimate hyperpolarizability, only the results obtained using the range-separated functionals CAM-B3LYP and *ω*B97-XD have been considered hereafter.

#### 3.2.4. Nonlinear optical properties (SHG, EOPE, DCSHG and OKE)

The NLO properties investigated in this work were predicted at the popular Nd:YAG laser frequency of 0.08242 arb. units using the range-separated functionals CAM-B3LYP and *ω*b97-XD together with the Ahlrich Def2-TZVPP basis set. Def2-TZVPP was chosen because larger polarized basis sets with electron correlation contributions give accurate estimates of electronic (hyper)polarizabilities [28]. The dynamic first hyperpolarizabilities of the molecules corresponding to EOPE [*β*(−*ω;ω,0*)] and SHG [*β*(*−2ω;ω,ω*)] are listed in table 6.

**Table 6.** First hyperpolarizabilities ( × 10^−30^ esu) of EMAB and its derivatives for SHG and EOPE at 1064 nm.

molecule	CAM-B3LYP/Def2-TZVPP					*ω*B97-XD/Def2-TZVPP				
	*β_x_*	*β_y_*	*β_z_*	*β_ztot_*	*β*_||_	*β_x_*	*β_y_*	*β*	*β_ztot_*	*β*_||_
SHG										
EMAB	55.5	6.6	−2.1	56.0	20.4	51.0	6.5	−2.1	51.5	19.0
A	−117	5.6	1.6	117	66.4	−106	5.6	1.3	106	59.9
B	168	−10.4	2.7	169	87.0	145	−9.5	2.4	146	72.1
C	91.4	−5.7	2.8	91.6	33.4	79.6	−5.4	2.6	79.8	28.6
D	117	−7.4	2.4	117	49.9	104	−7.1	2.3	104	44.4
PNA	0.3	0.8	0.0	0.8	0.4	0.3	0.7	0.0	0.7	0.4
EOPE										
EMAB	38.9	5.2	−1.6	39.3	14.0	36.2	5.1	−1.6	36.6	13.3
A	−74.2	4.4	0.7	74.3	41.9	−68.2	4.4	0.5	68.4	38.6
B	99.5	−6.8	1.7	99.8	51.6	88.6	−6.5	1.6	88.8	44.2
C	58.1	−4.2	1.9	58.3	21.0	51.7	−4.04	1.9	51.9	18.3
D	72.8	−5.2	1.7	73.1	30.8	66.1	−5.2	1.6	66.3	28.0
PNA	0.28	0.5	0.0	0.6	0.3	0.3	0.5	0.0	0.6	0.3

As expected, B and EMAB exhibit the highest and lowest SHG and EOPE capabilities respectively, at both levels of theory. Furthermore, the SHG and EOPE values for the molecules studied are respectively about 70–200 and 70–170 times greater than those of PNA, indicating their suitability as materials for second harmonic generation and electro-optic Pockels effect. It is clear from table 6 that substitution of the methoxy group in EMAB with stronger electron donors improves the molecule's SHG and EOPE capabilities. According to the values of *β_tot_* and |*β*|, EMAB and its derivatives are more likely to undergo SHG than EOPE. Among the compounds studied, B is the most promising SHG and EOPE chromophore.

The dynamic second-order hyperpolarizabilities corresponding to DCSHG and OKE are listed in table 7.

**Table 7.** Second hyperpolarizabilities ( × 10^−30^ esu) of EMAB and its derivatives for DCSHG and OKE at 1064 nm.

molecule	CAM-B3LYP/Def2-TZVPP					*ω*B97-XD/Def2-TZVPP				
	*γ_x_*	*γ_y_*	*γ_z_*	*γ_tot_*	〈*γ*〉	*γ_x_*	*γ_y_*	*γ_z_*	*γ_tot_*	〈*γ*〉
DCSHG										
EMAB	168	1.9	1.7	168	172	150	2.3	2.0	150	155
A	345	1.9	2.8	345	351	268	2.5	3.0	268	274
B	592	1.6	1.9	592	596	487	2.1	2.2	487	492
C	435	1.9	1.7	435	439	366	2.4	2.1	366	372
D	477	1.8	1.8	477	481	404	2.3	2.1	404	409
EOKE										
EMAB	107	1.7	1.6	107	110	97.4	21	1.9	97.5	101
A	174	2.0	2.3	174	179	15.5	2.4	2.7	155	161
B	307	1.4	1.7	307	310	265	1.9	2.1	265	270
C	248	1.6	1.6	248	252	217	2.1	1.9	217	221
D	265	1.6	1.6	265	268	233	2.0	2.0	233	237

According to the values, EMAB and its derivatives are significantly better NLO molecules than PNA. In increasing order of 〈*γ*〉, the molecules are ranked as follows: EMAB < D < C < A < B. Clearly, and as earlier mentioned, the derivatives of EMAB studied herein are better NLO molecules than EMAB. Moreover, B is the most promising NLO chromophore among the derivatives. It is equally worthy of note that although both range separated functionals give similar results for hyperpolarizabilities, *ω*B97-XD has been shown (in a benchmark study) to perform better than CAM-B3LYP in computing hyperpolarizabilities in D-*π*-A systems [4]

### 3.3. Frontier molecular orbital analysis

The energy difference (denoted *E*_gap_ herein) between the highest occupied molecular orbital (HOMO) and the lowest unoccupied molecular orbital (LUMO) is a key parameter when predicting polarizability and reactivity in molecules [6]. Molecules with a small band-gap are more polarizable and chemically more reactive [54]. The *E*_gap_ values and some NLO parameters are compared in table 8.

**Table 8.** Some selected values of quadratic ( × 10^−30^ esu), cubic ( × 10^−36^ esu) NLO properties and energy gaps of EMAB and its derivatives calculated at 1064 nm and CAM-B3LYP/Def2-TZVPP and *ω*B97-XD/Def2-TZVPP levels of theory respectively.

molecule	*E*_gap_ (eV)	β(0, 0, 0)	γ(0, 0, 0, 0)	*β*_vec_ at 1064	*β*_vec_ at 532	SHG	EOPE	DCSHG	EOKE
CAM-B3LYP/Def2-TZVPP									
EMAB	6.4224	33.8	88.4	34.0	595	56.0	39.3	168	107
A	5.9906	61.6	138	111	−477	117	74.3	345	174
B	5.7217	80.7	237	145	−286	169	99.8	592	307
C	5.9903	48.5	198	55.7	−125	91.6	58.3	435	248
D	5.8774	60.2	209	83.2	−218	117	73.1	477	265
*ω*B97-XD/Def2-TZVPP									
EMAB	7.5552	31.7	81.1	31.7	401	51.5	36.6	150	97.5
A	7.1073	57.2	124	99.8	−636	106	68.4	268	155
B	6.8420	73.0	208	120	−281	146	88.8	487	265
C	7.1182	43.7	175	47.6	−143	79.8	51.9	366	217
D	6.9952	55.4	186	73.9	−226	104	66.3	404	233

It is conceivable from table 8 that NLO activity is inversely proportional to *E*_gap_. For instance, molecule B which possesses the highest predicted NLO activities has the least *E*_gap_ value at both levels of theory. Indeed, the *E*_gap_ values follow the order: B < D < C < A < EMAB, which is in sharp contrast to the NLO activity ranking. This further confirms B as the best NLO chromophore among the molecules studied.

**Figure 6.**
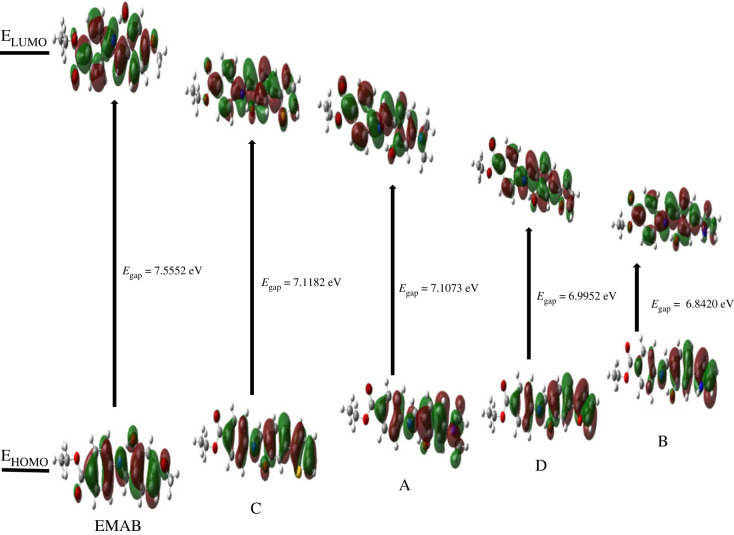
Frontier molecular orbitals of EMAB and its derivatives studied, obtained at *ω*B97-XD/Def2tzvpp level of theory.

The molecular orbital surfaces of the HOMOs and LUMOs are displayed in figure 6 as visualized using GaussView 6.0 [31]. It can be seen that HOMO → LUMO electronic excitation results in donor-to-acceptor electron density shift that enhances ICT in the molecules.

### 3.4. Time-dependent density functional theory study

In the realm of nonlinear optics, an undesirable effect, known as transparency/efficiency trade-off usually leads to reduced device efficiency. Molecules possessing high optical transparencies in the visible region suffer very low optical losses hence, transparency–nonlinearity trade-offs are highly minimized. Unfortunately, factors that improve the NLO activities of molecules, such as longer *π*-conjugated linkers and stronger donor/acceptor groups, usually lead to a red shift of electronic absorption maxima. This red shift of the absorption maximum encourages transparency/efficiency trade-off. Strong absorption of light in the visible region of the electromagnetic (EM) spectrum limits NLO applications of materials [55]. To determine the likelihood of this undesirable phenomenon in the investigated molecules, their UV-Vis absorption spectra were predicted using the TD-DFT method. The computed absorption wavelengths (*λ*_max_) and oscillator strengths (*f*) based on the first 10 vacant molecular orbitals, are listed in table 9.

**Table 9.** Absorption wavelengths (*λ*, nm), highest oscillator strengths (*f*), and main transitions of EMAB and its derivatives, calculated at CAM-B3LYP/Def2-TZVPP and *ω*B97-XD levels of theory by the TD-DFT method.

molecule	level of theory	*λ*_max_	oscillator strength	main assignment^a^
EMAB	*ω*B97-XD	311.94	0.9586	(0.65440) H → L
	CAM-B3LYP	314.48	0.9748	(0.66484) H → L
A	*ω*B97-XD	327.98	1.2809	(0.64387) H → L
	CAM-B3LYP	331.98	1.2857	(0.65891) H → L
B	*ω*B97-XD	341.82	1.3524	(0.61993) H → L
	CAM-B3LYP	347.27	1.3667	(0.61993) H → L
C	*ω*B97-XD	334.73	1.1649	(0.61993) H → L
	CAM-B3LYP	338.76	1.1987	(0.61993) H → L
D	*ω*B97-XD	339.04	1.2404	(0.61993) H → L
	CAM-B3LYP	343.11	1.2691	(0.61993) H → L

^a^H stands for HOMO and L stands for LUMO.

The results from the table demonstrate that the maximum absorption wavelengths of all investigated molecules fall within the UV region (100–400 nm) of the EM spectrum. This unequivocally shows that the molecules have high optical transparencies in the visible region, and are therefore expected to suffer minimal optical losses, leading to reduced transparency–nonlinearity trade-off. We notice that HOMO → LUMO electronic transitions contribute more than 60% of the maximum absorption bands. As can be seen, substitution leads to a red shift of the absorption maxima, by the well-established fact that substituting D-π-A systems with stronger donors causes more electrons to be pushed into the molecule, thereby increasing *λ*_max_ [56]. Fortunately, the red-shifted bands remain in the UV region. Interestingly, similar results have been obtained at both levels of theory. It is also interesting to note that the SHG wavelength (532 nm) of the Nd:YAG laser (*λ* = 1064 nm) used in this study is far (below) from *λ*_max_ in all cases.

### 3.5. Density of states

**Figure 7.**
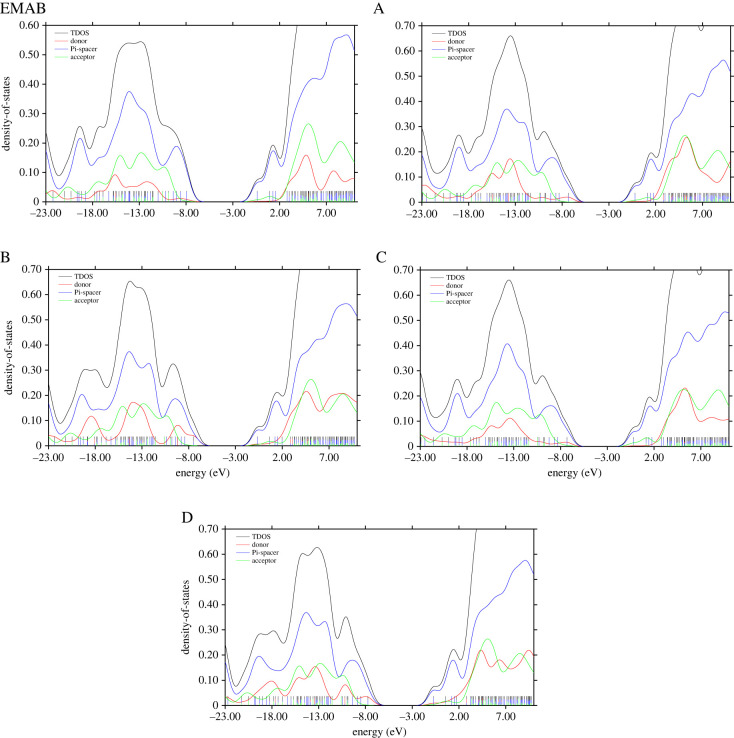
Density of states (DOS) plot for EMAB and its designed derivatives.

In a bid to complement the results obtained from frontier molecular orbital analysis, the density of states (DOS) plots for all the molecules under investigation have been generated from Gaussian output files obtained at the *ω*B97-XD/Def2TZVPP level of theory. DOS essentially refers to the number of different states in unit energy interval that can be occupied by electrons. It is interesting to note that DOS provides useful information regarding the delocalization of electrons in the frontier molecular orbitals [57]. For clarity, the total density of states (TDOS) has been plotted along with the partial contributions from the donor, π-spacer and acceptor for each D-π-A chromophore studied (figure 7). The DOS plots were generated with the aid of Multiwfn-3.8 [32] using the Hirshfeld method as implemented therein.

From figure 7, the DOS maps are characterized by a band gap of about 7.00 eV that separates the HOMO (at approx. −7.50 eV) from the LUMO (at approx. −0.50 eV), which is in perfect agreement with the values obtained from the frontier molecular orbital analysis, as reported in electronic supplementary material, table S7. The DOS plots further indicate that the HOMO in each case is predominantly contributed by the donor and the π-spacer, but the contribution from the former increases with donor strength. Accordingly, the greatest donor contribution to the HOMO is observed in molecule B, which has been earlier found to exhibit the greatest NLO activity. On the other hand, the π-linker and somewhat the acceptor are the main contributors to the LUMOs [58]. It is therefore clear from the foregoing observations that HOMO → LUMO electronic excitations in EMAB and its derivatives studied can lead to ICT from the donor through the π-linker to the acceptor, which is likely to enhance their NLO properties.

### 3.6. Natural bond orbital analysis

Natural bond orbital (NBO) analysis has been reported as an efficient technique for determining bonding interactions and ICT in molecular species [1,58]. Besides the foregoing, this technique has been used to compute the natural charges on the atoms comprising the donor, acceptor and π-linker in each NLO chromophore investigated herein. The net natural atomic charge (NPA) on each of these moieties is reported in table 10.

**Table 10.** Natural atomic charges calculated at *ω*B97XD/Def2TZVPP level of theory.

molecule	donor	π-spacer	acceptor
EMAB	−0.1605	0.1643	−0.0038
A	0.0179	−0.0096	−0.0083
B	0.0577	−0.0550	−0.0027
C	0.0267	−0.0252	−0.0015
D	0.0355	−0.0333	−0.0022

It is noteworthy that the natural charges and natural bonding orbitals (NBOs) were computed at the *ω*B97-XD/Def2TZVPP level of theory using the NBO 3.1 module [59] embedded in Gaussian 09. The natural atomic charges on individual atoms are provided in electronic supplementary material, table S8.

Results from table 10 reveal net negative charges on the donor and acceptor moieties in EMAB, suggesting that EMAB is a somewhat A-π-A species. The scenario in the substituted derivatives of EMAB is different, since the net charge on their donor moieties is positive. Arguably, substitution transforms the A-π-A configuration of EMAB into D-π-A, thereby enhancing its NLO activity in each case studied. Indeed, the net positive charge on the donors and net negative charge on the acceptors affirm their electron donating and accepting abilities respectively, through the π-bridge [58]. However, the slight negative charge on the π-bridge in EMAB's derivatives indicates that the bridge may cause minimal charge trapping. On the basis of NPA (the magnitude of the net positive charge), electron donor strength increases in the order: EMAB < A < C < D < B, which makes the designed molecules more efficient NLO chromophores than EMAB, as shown by their NLO properties earlier computed. It follows from the preceding ranking that the pyrrole substituent is the best donor among those investigated.

To decipher the charge transfer patterns in the molecules studied, some important conjugative and hyper-conjugative NBO interactions computed are listed in table 11, alongside their stabilization energies (*E*^(2)^) calculated based on the second-order perturbation theory. Additional donor–acceptor NBO interactions for EMAB and its derivatives are provided in electronic supplementary material, tables S9–S11.

**Table 11.** Some selected electronic transitions and stabilization energies for EMAB, A, B, C and D, calculated at *ω*B97XD/Def2TZVPP level of theory.

molecule	donor *i*	type	acceptor *j*	type	*E*^(2)^ kcal mol^−1^
EMAB	C25-C27	π	C30-O31	π*	26.02
	O32	LP	C30-O31	π*	66.22
	C3-C4	π	C17-N18	π*	36.21
	C17-N18	π	C20-C22	π*	13.81
	C1-C2	π	C5-C6	π*	47.61
	C5-C6	π	C3-C4	π*	51.95
	C3-C4	π*	C17-N18	π*	194.52
	C20-C22	π*	C21-C23	π*	255.97
	O10	LP	C5-C6	π*	43.91
A	C24-C26	π	C29-O30	π*	26.67
	O31	LP	C29-O30	π*	65.65
	C16-N17	π	C19-C21	π*	15.19
	C19-C21	π	C20-C22	π*	263.9
	C4-C5	π	C3	LP*	36.8
	C3	LP	C16-N17	π*	83.9
	C3	LP	C4-C5	π*	96.26
	N39	LP	C6	LP*	128.76
B	C20-C22	π	C25-O26	π*	25.89
	O27	LP	C25-O26	π*	66.31
	C2-C3	π	C12-N13	π*	33.25
	C12-N13	π	C15-C17	π*	13.81
	C2-C3	π*	C12-N13	π*	206.8
	C35-C36	π	C1-C6	π*	20.5
	N42	LP	C35-C36	π*	54.8
C	C20-C22	π	C25-O26	π*	25.7
	O27	LP	C25-O26	π*	66.48
	C2-C3	π	C12-N13	π*	31.72
	C12-N13	π	C15-C17	π*	13.55
	C2-C3	π*	C12-N13	π*	203.38
	C35-C36	π	C1-C6	π*	15.52
	S39	LP	C35-C36	π*	36.26
D	C20-C22	π	C25-O26	π*	25.79
	O27	LP	C25-O26	π*	66.4
	C2-C3	π	C12-N13	π*	32.29
	C12-N13	π	C15-C17	π*	13.62
	C2-C3	π*	C12-N13	π*	212.78
	C35-C36	π	C1-C6	π*	18.86
	O42	LP	C35-C36	π*	42.16

For each donor (*i*) and acceptor (*j*) NBO pair, the value of *E*^(2)^ was estimated according to equation (3.1).

3.1 $$E^{\left(2\right)}=q_{i}\frac{{\left(F_{i,j}\right)}^{2}}{\varepsilon_{j}-\varepsilon_{i}},$$

where *q_i_* is the orbital occupancy, ε*_j_* and ε*_i_* are diagonal elements, *F_i_*_,*j*_ is the off-diagonal NBO Fock matrix element. The larger the value of *E*^(2)^, the more intensive is the interaction between an electron donor NBO and an acceptor NBO. Note that electron density delocalization between an occupied NBO (bonding or lone pair) and a formally unoccupied NBO (antibonding or Rydberg) corresponds to a stabilizing interaction [60].

The results show that several conjugative interactions that result in charge transfer from the π-spacer to the electron acceptor in the push-pull molecules studied have been observed. These include: π(C25-C27) → π*(C30-O31) and LP(O32) → π*(C30-O31) for EMAB with stabilization energies 26.02 and 66.22 kcal mol^−1^, π(C24-C26) → π*(C29-O30) and LP(O31) → π*(C29-O30) for A with energies 26.67 and 65.65 kcal mol^−1^, as well as π(C20-C22) → π*(C25-O26) for B, C and D with energies 25.89, 25.7 and 25.79 kcal mol^−1^, respectively. Furthermore, the interaction LP(O27) → π*(C25-O26) for B, C and D with stabilization energies 66.31, 66.48 and 66.4 kcal mol^−1^, respectively, is also found to be one of the major contributors to the *π*-spacer → electron-acceptor ICT. In all cases above, the acceptor NBO is localized in the ester group, thus confirming the group's role as an electron acceptor in the push-pull molecules studied.

Several NBO interactions describing π-conjugation and electron delocalization within the π-linker have been identified. For EMAB, these interactions include: π(C3-C4) → π*(C17-N18), π(C17-N18) → π*(C20-C22), π(C1-C2) → π*(C5-C6) and π(C5-C6) → π*(C3-C4) with energies 36.21, 13.81, 47.61 and 51.95 kcal mol^−1^ respectively and π*(C3-C4) → π*(C17-N18) and π*(C20-C22) → π*(C21-C23) with energies 194.52 and 255.97 kcal mol^−1^. For A, the interaction comprises π(C16-N17) → π*(C19-C21), π*(C19-C21) → π*(C20-C22), π(C4-C5) → LP*(C3), LP(C3) → π*(C16-N17) and LP(C3) → π*(C4-C5) with energies 15.19, 263.9, 36.8, 83.9 and 96.26 kcal mol^−1^, respectively. For the molecules B, C and D, the interactions are common and include: π(C2-C3) → π*(C12-N13) with energies 33.25. 31.72, and 32.29 kcal mol^−1^, π(C12-N13) → π*(C15-C17) with energies 13.81, 13.55 and 13.62 kcal mol^−1^, and π*(C2-C3) → π*(C12-N13) having energies 206.8, 203.38 and 212.78 kcal mol^−1^ for B, C and D, respectively. Owing to these intensive (hyper)conjugative interactions, the π-linker in the present molecules is extensively π-conjugated and should immensely facilitate charge transfer donor-acceptor ICT.

For the donors in the push-pull structures under investigation, the main donor → π-spacer charge transfer interactions for EMAB and molecule A are LP(O10) → π*(C5-C6) and LP(N39) → LP*(C6) with energies 43.91 and 128.76 kcal mol^−1^ respectively. The interaction π(C35-C36) → π*(C1-C6) is common to B, C and D with stabilization energies 20.5, 15.52 and 18.86 kcal mol^−1^, respectively. Finally, the interactions LP(N42) → π*(C35-C36), LP(S39) → π*(C35-C36) and LP(O42) → π*(C35-C36) had energies 54.8, 36.26 and 42.16 kcal mol^−1^, respectively for B, C and D. Thus substitution with better donors enhances charge transfer especially when the pyrrollyl donor is used.

## 4. Concluding remarks

Organic molecules are increasingly used as the building blocks of NLO materials owing to their fast NLO responses, easy synthesis, cost effectiveness and easy growth into high-quality crystals. The Schiff base molecule ethyl 4-[(*E*)-(2-hydroxy-4-methoxyphenyl)methyleneamino]benzoate (EMAB) possesses the structural characteristics of a good NLO molecule, but its NLO properties have not been investigated to date. In the present study, the DFT quantum chemical method has been used to study the NLO properties of EMAB and its derivatives obtained through substitution of its methoxy group with stronger electron donors. Furthermore, the electronic absorption spectra of the molecules have been studied via the TD-DFT approach. Several functionals (B3LYP, CAM-B3LYP, M06-2X and *ω*B97-XD) and basis sets (6-31 + G**, 6-311 + + G** and Def2-TZVPP) were used. Prior to the NLO studies, conformational analysis was performed to determine the most stable conformer of EMAB. From the results obtained, the static first and second hyperpolarizabilities of the molecules (31.7–86.5 × 10^−30^ and 84.4–273 × 10^−36^ esu, respectively) as well as the dynamic NLO properties are at least 28 times larger than those of the prototype NLO molecule, *para*-nitroaniline. The maximum absorption wavelengths of the molecules fall within the UV region of the electromagnetic spectrum. Therefore, EMAB and its derivatives studied are promising candidates for the fabrication of NLO devices that are unlikely to suffer significant transparency–nonlinearity trade-offs. Interestingly, the frontier molecular orbital studies, supported by NBO and DOS revealed effective charge transfer within each molecule from the HOMOs to the LUMOs. Furthermore, the derivatives of EMAB with stronger electron donors have been found to possess better NLO properties than EMAB. Accordingly, the NLO activity of EMAB can be improved through substitution of its methoxy group with stronger electron donors, particularly the pyrrolyl donor group.

## Data Availability

The data are available from the Dryad Digital Repository: https://doi.org/10.5061/dryad.t1g1jwt5n [61]. The data are provided in electronic supplementary material [62].
